# Modulation of plasma complement by the initial dose of epirubicin/docetaxel therapy in breast cancer and its predictive value

**DOI:** 10.1038/sj.bjc.6605909

**Published:** 2010-09-28

**Authors:** A Michlmayr, T Bachleitner-Hofmann, S Baumann, M Marchetti-Deschmann, I Rech-Weichselbraun, C Burghuber, U Pluschnig, R Bartsch, A Graf, R Greil, G Allmaier, G Steger, M Gnant, M Bergmann, R Oehler

**Affiliations:** 1 Department of Surgery, Medical University of Vienna, A-1090, Vienna, Austria; 2 Institute of Chemical Technologies and Analytics, Vienna University of Technology, A-1060, Vienna, Austria; 3 Bender MedSystems GmbH., A-1030, Vienna, Austria; 4 Department of Internal Medicine-I, Medical University of Vienna, A-1090, Vienna, Austria; 5 Section of Medical Statistics, Medical University of Vienna, A-1090, Vienna, Austria; 6 3rd Medical Department, Private Medical University Hospital Salzburg, A-5020, Salzburg, Austria

**Keywords:** breast cancer, response to therapy, epirubicin, docetaxel, complement system

## Abstract

**Background:**

Despite the widespread use of neoadjuvant chemotherapy in breast cancer patients, prediction of individual response to treatment remains an unsolved clinical problem. Particularly, administration of an inefficient chemotherapeutic regimen should be avoided. Therefore, a better understanding of the molecular mechanisms underlying response to neoadjuvant chemotherapy is of particular clinical interest. Aim of the present study was to test whether neoadjuvant chemotherapy with epirubicin/docetaxel induces early changes in the plasma proteome of breast cancer patients and whether such changes correlate with response to therapy.

**Methods:**

Plasma samples of 25 breast cancer patients obtained before and 24 h after initiation of epirubicin/docetaxel-based neoadjuvant chemotherapy were analysed using two-dimensional differential gel electrophoresis (2D-DIGE). Protein spots found to be differentially expressed were identified using mass spectrometry and then correlated with the pathological response after six cycles of therapy. Markers identified in a discovery set of patients (*n*=12) were confirmed in an independent validation set (*n*=13).

**Results:**

2D-DIGE revealed 33 protein spots to be differentially expressed in response to chemotherapy, including the complement factors C1, C3 and C4, inter-*α*-trypsin inhibitor, *α*-1-antichymotrypsin and *α*-2-Heremans-Schmid glycoprotein (AHSG). With respect to cytokines, only interleukin (IL)-6, IL-10 and soluble intracellular adgesion molecule 3 (sICAM3) were minimally modulated. Moreover, two protein spots within the complement component C3 significantly correlated with response to therapy.

**Conclusion:**

We have identified acute phase proteins and the complement system as part of the early host response to epirubicin/docetaxel chemotherapy. As complement C3 cleavage correlates with the efficacy of docetaxel/epirubicin-based chemotherapy, it has the potential as an easily accessible predictive biomarker.

Neoadjuvant chemotherapy has become the standard treatment for patients with locally advanced breast cancer or patients with operable breast cancer in which breast-conserving surgery is not possible or unlikely to result in a satisfactory cosmetic outcome. However, the individual response to therapy is widely variable. Currently, it is not possible to accurately predict the response in an individual patient. The only response markers available—albeit with a low accuracy—are low or absent hormone receptor status, high grade and non-lobular invasive histology (Kaufmann *et al*, 2007). As a consequence, more accurate and clinically useful predictors of response for breast cancer patients receiving neoadjuvant chemotherapy need to be identified.

Ample evidence suggests that the efficacy of chemotherapy is related both to the molecular profile of the tumour (Potti *et al*, 2006), as well as the host response to therapy (Zitvogel *et al*, 2008). The introduction of profiling technologies has enabled genome- or proteome-wide searches for predictive and prognostic biomarkers in pre-therapeutic biopsies of tumour and surrounding host tissue. However, only a few predictive marker sets have yet been successfully validated for routine use in the clinic (van de Vijver *et al*, 2002; Paik *et al*, 2004). A significant limitation of the above approaches is that only a single point in time is taken into consideration. Thus, dynamic changes during the course of therapy, which may reflect treatment-associated tumour/host changes correlating with response to therapy, are not adequately addressed.

Preliminary studies have shown that radio- or chemotherapy-induced changes of apoptotic pathways can be detected by immunochemistry as early as 48 h after initiation of treatment (Stift *et al*, 2003) and correlated with therapeutic efficacy (Stearns *et al*, 2003, p. 23). Further support for this observation comes from a recent study in patients with rectal cancer who received 5 weeks of cetuximab-based neoadjuvant therapy (Debucquoy *et al*, 2009). The authors analysed the levels of 96 different proteins in plasma samples taken before and one day after application of the initial chemotherapeutic dose. Therapy-induced changes in expression of six proteins after the initial dose were found to predict the occurrence of local recurrences and/or distant metastases after the end of therapy.

On the basis of the above results, we hypothesised that the immediate response of tumour and/or host cells to neoadjuvant chemotherapy results in distinct early changes of the plasma protein composition. We also hypothesised that these early ‘host changes’ may correlate with response to treatment that could allow for early response prediction using easily accessible predictive markers: that is, patients who are predicted to be non-responders to neoadjuvant chemotherapy could either be switched early to a different, potentially more effective therapeutic regimen or, alternatively, be scheduled for upfront surgery.

In the present study, we performed serial proteomic analyses of the plasma protein composition of 25 breast cancer patients receiving neoadjuvant chemotherapy with epirubicin and docetaxel. Our results indicate that the initial dose of epirubicin and docetaxel leads to a significant modulation of the complement cascade, whereas the cytokine cascade is little affected. It is important that we were able to identify two complement isoforms the plasma levels of which correlated with response to therapy. If validated, our results could form an important basis for future complement-based response prediction systems in breast cancer patients receiving neoadjuvant chemotherapy.

## Materials and methods

### Patient characteristics

Between November 2005 and August 2008, 25 patients with biopsy-proven early or locally advanced breast cancer who were suitable for neoadjuvant chemotherapy were enrolled in this study (for patient characteristics see Table 1). The therapeutic regimen consisted of six cycles of neoadjuvant chemotherapy with epirubicin and docetaxel (every 3 weeks for a total duration of 18 weeks) with the addition of granulocyte colony stimulating factor (G-CSF) support. Blood samples were taken immediately before (T1) and 24 h after the initial dose of the therapy (T2). For assessment of response to chemotherapy, the tumour size upon radiological imaging before initiation of treatment (S1) was compared with the tumour size upon pathohistological examination (S2), using the World Health Organization criteria (Miller *et al*, 1981). Complete remission was defined as disappearance of all invasive tumours in the pathological specimen (‘pathological complete response’). Patients achieving a complete or partial remission were defined as responders, whereas patients with stable or progressive disease were defined as non-responders. The study was approved by the local ethics committee.

### Blood collection and sample preparation

Blood was collected in K_3_EDTA tubes (Vacutainer, Greiner Bio-One, Kremsmünster, Austria) and plasma was prepared within 60 min by centrifugation at 2400 **g** for 15 min. The upper 2/3 of the supernatant were taken, immediately shock frozen and were stored at −80 °C for 3–16 months. In a pilot experiment with four parallel gels per group we found no significant difference in the proteome analysis between samples that were stored for either 6 or 11 months. In addition, the storage time was nearly identical for samples taken from responders (3–15 months) and non-responders (5–16 months). The 12 most abundant plasma proteins (human serum albumin, immunoglobulin (Ig) G, *α*-1-antitrypsin, IgA, IgM, transferrin, haptoglobin, *α*-1-acid glycoprotein, *α*-2-macroglobulin, apolipoproteins A-I and A-II and fibrinogen) were removed from each sample using the ProteomeLab IgY12-LC2 proteome partitioning kit (Beckman Coulter, Fullerton, CA, USA). The remaining plasma proteins were precipitated using trichloroacetic acid as described (Zellner *et al*, 2005) and resolubilised in 2D sample buffer containing 7 M urea, 2 M thiourea, 4% 3-((3-cholamidopropyl)dimethylammonium)-1-propanesulfonate (CHAPS) and 25 mM Tris-HCl (pH 8.5). The protein concentration was determined using the Coomassie Plus Protein Assay Reagent (Pierce, Rockford, IL, USA).

### Proteomic analysis

Equivalents of 20 *μ*g of the two depleted plasma samples per patient (T1 and T2) were labelled alternating with fluorescent cyanine dye Cy3 or Cy5 according to the manufacturer's protocol (GE Healthcare, Uppsala, Sweden). In addition, a pool of an aliquot of all samples of all patients was labelled with Cy2 as an internal standard. Two samples derived from one patient and 20 *μ*g of internal standard were pooled and filled up with rehydration solution (7 M urea, 2 M thiourea, 4% CHAPS, 70 mM dithiothreitol, 1% ampholytes pH 4–7, bromophenol blue) to 450 *μ*l each. Isoelectric focusing was carried out on Ettan IPGphor Isoelectric Focusing Units (GE Healthcare) after passive sample rehydration in 24 cm Immobiline DryStrips pH 4-7 (GE Healthcare) until 30 kVh were reached. Then strips were equilibrated for 15 min in equilibration solution (6 M urea, 50 mM Tris-HCl (pH 8.8), 30% glycerol, 2% sodium dodecyl sulphate) containing 10 mg ml^−1^ dithiothreitol (Roche Diagnostics, Mannheim, Germany) and subsequently for 15 min in equilibration solution containing 25 mg ml^−1^ iodoacetamide (Sigma-Aldrich, St Louis, MO, USA). Strips were then applied on 9% polyacrylamide gels and electrophoresis was performed using the Ettan DALT six apparatus (GE Healthcare). Then gels were scanned as described previously (Winkler *et al*, 2008) using the Typhoon TRIO—Variable Mode Imager (GE Healthcare).

Spots were excised from gels and subjected to in-gel digestion using trypsin (proteomics grade, Roche Diagnostics). After ZipTip C18 purification the digest extracts were analysed on a matrix-assisted laser desorption ionisation time-of-flight mass spectrometer (CFRplus, Shimadzu Biotech Kratos Analytical, Manchester, UK) using the thin layer preparation technique (Vorm *et al*, 1994) with *α*-cyano-4-hydroxycinnamic acid (Sigma-Aldrich) as matrix. All mass spectra were recorded in the positive ion reflectron mode. After manual peak picking lists of monoisotopic m/z values were submitted to publically available, web-based versions of peptide mass fingerprint search engines (MASCOT (Perkins *et al*, 1999)), Aldente and ProFound (Zhang and Chait, 2000) using the NCBInr database (date: 20090214; taxonomy human: 221591 protein sequences). From the peptide mass fingerprints selected tryptic peptides were chosen for post source decay experiments. Protein identification was further confirmed by submitting m/z values of detected fragment ions to MASCOT. If post source decay experiments were not successful a protein was still considered as identified if at least two different search engines (MASCOT, ProFound or Aldente) listed the same protein after peptide mass fingerprint search and the protein was located at a reasonable location on the 2D gels. No special emphasis was laid on discrimination of protein isoforms.

### Western blot

Western blot of 2D gels was performed as described elsewhere (Zellner *et al*, 2005). Following primary antibodies were used: polyclonal chicken anti-human C3 (1 : 10 000) (Lee Biosolutions, St Lousi, MO, USA), polyclonal goat anti-human C4 (1 : 10 000) (Lee Biosolutions), murine monoclonal anti-human C4d (1 : 2000) (Quidel, San Diego, CA, USA). After washing, membranes were incubated for 1 h at room temperature with the corresponding Cy5 conjugated secondary antibodies (Cy5-conjugated donkey anti-mouse IgG (Fab fragment; 1 : 1000) (Jackson ImmunoResearch Laboratories, Suffolk, UK), Cy5-conjugated rabbit anti-chicken IgY (1 : 500) (Biomeda, Foster City, CA, USA), Cy5-conjugated donkey anti-goat IgG (Fab fragment; 1 : 500) (Jackson ImmunoResearch Laboratories) in 2% non-fat dry milk. To match the western blot images with the two-dimensional differential gel electrophoresis (2D-DIGE) images the gel image warping software Delta-2D (Decodon, Greifswald, Germany) was used.

### Immunologic analyses

C4d fragment enzyme immunoassay, in which the plasma samples were diluted 1 : 40, was performed according to the manufacturer's protocol (Quidel). Total levels of complement components C3 and C4 in plasma were determined using the assay kit N Antisera to Human Complement Factors (C3c, C4) (Dade Behring, Marburg, Germany), which is based on immunonephelometry. A Human Th1/Th2 Plex FlowCytomix kit (Bender MedSystems, Vienna, Austria) was used to quantify the plasma cytokine levels. The assay was performed according to the manufacturer's instructions. Beads were run on a FC500 flow cytometer (Beckman-Coulter, Miami, FL, USA) for data collection. Further quantification was performed using the FlowCytomix Pro 2.2 software (Bender MedSystems).

### Statistical analysis

Estimation of sample size for proteomic analysis of the discovery set was based on the results of our previous study on the biological variation in proteomic samples (Winkler *et al*, 2008). Using the same DIGE technology as in the present work to analyse blood samples from 20 healthy volunteers revealed a median variation *σ* over all proteins of 18%. In the present study, we monitored only mean protein expression changes (Δ) between T1 and T2 of at least 30%. This consequently corresponds to a minimal effect size *θ*=Δ/*σ*=0.3/0.18=1.7. On the basis of this *θ* we calculated the asymptotic power per hypothesis of the two-sided two-sample paired Student's *t-*test. The error of multiple testing was corrected using an asymptotic false discovery rate (according to (Benjamini and Hochberg, 1995)) of 5% targeted at 700 candidate proteins in which 25 of the proteins were effective. For a sample size of 12 per group the resulting power was 0.92, which was regarded as acceptable. In the validation set we were dealing with a much lower number of parameters (19 C3 and 16 C4 spots) in comparison to the discovery set (about 700 spots) with nearly the same sample size (*n*=13 *vs n*=12). Thus, the problem of multiple testing was much lower resulting in a considerably higher statistical power.

Quantitation of protein spots was accomplished using the DeCyder 2D software (version 6.0 from GE Healthcare). Protein spots outside the molecular weight range of 25–250 kDa were excluded from the analysis. Only protein spots that were found in at least 9 of 12 gels were included. A total of 688 distinct protein spots fulfilled these conditions. The paired ratio of the expression at the time T2 to the expression at T1 was assessed for each spot and the significance was calculated using a false discovery rate adjusted paired Student's *t*-test as described above.

For determination of the correlation of the effect of the initial dose with response to chemotherapy, the differences between protein expression values of time T2 and T1 were assessed for each spot. Student's *t*-test was used to compare these differences (T2–T1) between non-responders and responders. Differences in total protein levels of complement components C3, C4 and C4d were calculated using the paired Student's *t*-test. Receiver operating characteristic (ROC) curves were calculated using the SPSS software (Version 17.0, IBM Corporation, Armonk, NY, USA).

## Results

### Impact of neoadjuvant chemotherapy with epirubicin and docetaxel on plasma proteins

**Figure 1:**
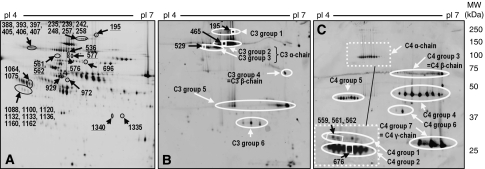
Two-dimensional gel electrophoresis of the plasma proteome. (**A**) Position of protein spots most significantly influenced by the initial dose of the therapy on a representative 2D-gel image. Their molecular identity is indicated in Table 2. The underlined numbers represent members of the complement system. (**B** and **C**) show 2D-western blot images using antibodies against complement component C3 (**B**) or C4 (**C**). The detail displayed in the punctured frame shows the signals obtained by incubation with monoclonal anti-C4d antibody. Only this antibody recognised the upper C4 *α*-chain. Protein spots indicated in rectangles represent complement isoforms that are significantly differentially influenced by the initial dose between responders and non-responders.

First we investigated whether the initial dose of the neoadjuvant chemotherapy given for breast cancer affects the plasma proteome at 24 h after initiation of therapy as assessed by (2D-DIGE). Patients receiving an epirubicin and docetaxel combination therapy in six cycles over 18 weeks were included into the study. Therefore, samples were taken from 12 patients (=discovery set) immediately before they received their initial dose (T1) and the next day (T2) before G-CSF was given. In total, 688 distinct protein spots on the 2D gel were analysed quantitatively in each sample. Expression levels of 33 protein spots were found to be significantly influenced by the chemotherapeutic initial dose by at least 30%. Figure 1A indicates their position on a representative 2D gel (see also Supplementary Data S1). The molecular identity of these spots has been revealed by mass spectrometry. Table 2 summarises the results. Acute phase proteins, such as inter-*α*-trypsin inhibitor, *α*-1-antichymotrypsin and *α*-2-HS glycoprotein, as well as three different representatives of the complement system (C1r, C3 and C4), were found to be influenced by the combination therapy. In addition, an intracellular protein, L-plastin, being upregulated in response to chemotherapy, was identified (for further details on the mass spectrometry experiments see Supplementary Data S2). The 2D-DIGE analysis indicated that chemotherapy influenced the plasma concentration of these protein species at the level of different isoforms concomitantly. This supports the relevance of our findings.

### Impact of epirubicin and docetaxel combination therapy on the plasma cytokine pattern

We further analysed the levels of 17 different cytokines in the plasma samples, as a number of human and animal data suggest a cytokine response to chemotherapy. The results are summarised in Table 3 (upper panel). Seven cytokines, including the pro-inflammatory factors tumour necrosis factor-*α*, INF-*γ* and interleukin (IL)-1*β*, were below the detection limit before initiation of chemotherapy and remained undetectable after the initial dose of chemotherapy. The remaining 10 cytokines could be detected at both times. Only three of them showed significant therapy-induced expression changes: The plasma levels of pro-inflammatory IL-6, as well as of anti-inflammatory IL-10, were mildly increased during treatment, whereas the levels of soluble intracellular adgesion molecule 3 (sICAM3) were mildly decreased. However, the absolute plasma levels of all measured cytokines were at all times very low and always in a range observed for healthy volunteers.

### Therapy-induced concentration changes of complement factors C3 and C4

As a major finding of the 2D-DIGE analysis was the modulation of complement C3 and C4 factors, we next investigated whether complement modulation could also be monitored by standard assays. In a nephelometric assay for total plasma levels of C3 and C4, we found that plasma levels of both complement components showed a significant decrease in response to the initial chemotherapeutic dose, suggesting activation of the cascade (Table 3, lower panel). Therefore, in addition, we determined the amount of the C4d activation fragment by enzyme-linked immunosorbent assay. It is surprising that, in the latter assay we found a significant decrease in C4d fragment in response to chemotherapy. To validate these results, the complement factors were additionally analysed in an independent validation set, including 13 patients (see also Table 1). We observed largely identical absolute plasma levels, as well as a therapy-induced decrease in C3, C4 and C4d in the validation set as in the discovery set of patients. This confirms that the combination therapy influences the complement cascade within one day.

### Response prediction by early changes in plasma levels of complement factors C3 and C4

Next, we sought to investigate whether the chemotherapy-induced changes of complement components C3 and C4 can be correlated with the final response after six cycles of chemotherapy 18 weeks later. To do so, the patient population was subdivided in responders and non-responders. Response to chemotherapy was assessed by comparison of tumour size as assessed by radiological imaging before therapy (S1) with tumour size upon pathohistological examination (S2; see also Table 1). The discovery set contained six responders and six non-responders. The plasma levels of total C3, as revealed by the routine nephelometric method, decreased to a similar extent in both responders and non-responders (by 17 mg per 100 ml and 19 mg per 100 ml, respectively). Similarly, no difference between responders and non-responders regarding total C4 plasma levels was observed (4.6 mg per 100 ml *vs* 5.5 mg per 100 ml). The validation set was composed of six responders and seven non-responders. Here, again no significant difference was found for total C3 and total C4.

Activation of the complement system leads to proteolytic cleavage of the complement factors into smaller fragments. Clearly, measurement of total C3 and C4 levels cannot detect changes in the ratio between different fragments and isoforms of these factors. Western blot analysis of 2D-gels revealed that the plasma contains numerous isoforms of C3 and C4 (Figures 1B and C). The various spots were found to be ordered into groups on the 2D western blot membrane. According to their molecular weight and pI-value we could identify isoform groups belonging to the C3 *α*-chain (113 kDa), C3 *β*-chain (71 kDa), C4 *α*-chain (84 kDa), C4 *β*-chain (72 kDa), as well as the C4 *γ*-chain (33 kDa). The comparison of the positions of the chemotherapy-affected complement spots indicated in Figure 1A (underlined numbers) with the position of the C3 and C4 spots shown in Figures 1B and C suggests that epirubicin/docetaxel chemotherapy mainly affects the levels of C3 and C4 groups with a molecular weight above 75 kDa. This includes the *α*-chains of C3 and of C4, as well as C3 group 1, which likely represents the non-cleaved full length C3 protein. The *β* and *γ* chains, however, remained largely unaffected by chemotherapy. Next, we investigated whether there is a correlation with the final response to therapy at the level of single isoforms. Therefore, we calculated for all individual 19 C3 and 16 C4 spots (with a molecular weight above 75 kDa) the difference in their plasma levels between T2 and T1 and subdivided the results according to the response after completion of chemotherapy. First, the calculations were made for the discovery set. As shown in Table 4, the chemotherapy-induced changes in plasma levels of four C3 isoforms and three C4 isoforms were found to be significantly different between responders and non-responders. Specifically, in the group of non-responders the mean plasma level of the C3 group 1 spot 195 increased by 41.5% after the initial chemotherapy dose. This increase was significantly stronger in comparison to the responder group. In contrast, the C3 *α*1 spot 465 decreased in the non-responders, whereas it remained nearly unchanged in the responders. To confirm these data, we performed the same analysis in the validation set. The initial dose-induced plasma level changes of the above spots 195 and 529 showed again an association with the final response to therapy with a *P*-value of 0.047 for spot 195 and a borderline significant *P*-value of 0.054 for spot 529. Calculation of the combined significance for both the discovery and validation set taken together revealed highly significant *P*-values of 0.013 and 0.005, respectively. This strongly suggests that the immediate response of the plasma levels of these two C3 isoforms correlates with the final response of the tumour to chemotherapy.

Spot 529 was clearly assigned to the C3 *α*–chain, whereas spot 195 likely corresponds to non-cleaved C3. Correspondingly, the two spots changed contrariwise in response to chemotherapy: although spot 195 decreased in the group of responders, spot 529 increased (see Table 4). To estimate the predictive value of these changes, further calculations were made. We calculated the difference of the treatment-induced expression changes of the two spots in each patient (529–195). The result was used to calculate a receiver operating characteristics (ROC) of this score. The analysis revealed for the discovery set an area under the curve of 0.917 and for the validation set an area under the curve of 0.944. The area under the curve over all 25 samples was 0.908 (with a standard error of 0.063 and an asymptotic significance of 0.001). The 529-195 score was able to distinguish between responders and non-responders with a sensitivity of 0.80 and a specificity of 0.83. This suggests that the way in which complement factor C3 is processed in response to the initial dose of epirubicin/docetaxel combination therapy is indicative for the effect of the therapy on the tumour and could be useful to predict the outcome of treatment.

## Discussion

In the present study we have found that epirubicin/docetaxel chemotherapy leads to a modulation of the complement cascade, as well as the acute phase proteins inter-*α*-trypsin inhibitor, *α*-1-antichymotrypsin and *α*-2-HS glycoprotein. These effects were observed 24 h after the initial dose of therapy allowing for a very early evaluation of the body's response to treatment. Time course experiments revealed that most of these expression changes remained stable on day 2 and 3, but were undetectable at day 14 (data not shown). The complement system is affected by epirubicin/docetaxel chemotherapy at the level of different components of the cascade, as well as at the level of different isoforms of these components. This corroborates the significance of the results. It is important that a differential modulation of two C3 isoforms between responders and non-responders was observed in a training set of 12 patients which could be confirmed in an independent validation set of 13 patients. Our data highlight the role of complement as an important host response factor that could be used for early identification of non-responders to neoadjuvant chemotherapy in breast cancer patients.

Despite a growing body of evidence illustrating the complement system as an important factor in cancer patients, the complement system has so far achieved little attention in clinical oncology. Previous proteomic studies found increased levels of different components of the complement system in sera of patients with colorectal cancer (Habermann *et al*, 2006; Ward *et al*, 2006) and in plasma of patients with breast cancer (Nakshatri *et al*, 2009). A study in patients with pancreatic cancer revealed that preoperative plasma levels of nine proteins, including complement factors C1q-B, C3 and C4A correlated with the 1-year disease-free survival (Lin *et al*, 2006). Moreover, a panel of five serum proteins, including complement factor C3a was able to predict the 5-year metastasis-free survival in breast cancer patients (Goncalves *et al*, 2006). However, complement component C3a was found to lack specificity for early diagnosis of breast cancer, limiting its utility as a stand-alone tumour marker (Li *et al*, 2005).

Usually, complement is seen as an immunological guardian against pathogens and damaged cells. In this line complement is thought to promote antibody-dependent cytotoxicity in the cancer setting (Macor and Tedesco, 2007). However, recent murine experiments have also indicated that the activation of C5a, which is activated by C3, leads to immunosuppression of tumour-infiltrating monocytes (Markiewski *et al*, 2008). In this way C5a promotes murine tumour growth as tumours frequently modulate host response signals from the microenvironment for their own advantage. Furthermore, cancer cells have been found to be more resistant to complement-mediated lysis and they use this attribute to set up a locally immunosuppressive environment (Loveland and Cebon, 2008).

In the present study, we describe for the first time a modulation of the complement cascade by a specific chemotherapy in breast cancer patients. We demonstrate a reduced level of total C3 and C4 that is suggestive of complement activation in response to chemotherapy, even though we did not find an increase of the complement activation marker C4d in our samples. We still favour the interpretation of our data as complement activation, as there is also evidence for several bypass pathways of complement activation (Li *et al*, 2007). Activation might be induced by chemotherapy-induced apoptosis of tumour cells. Corroboration for this hypothesis comes from the fact that complement factors are involved in the removal of apoptotic cells. Complement factors C1q, C3 and C4 have been described to bind specifically to apoptotic cells (Paidassi *et al*, 2008). Such marked cells are then recognised and eliminated by macrophages in a process involving C1q binding to cell surface expressed calreticulin (Vandivier *et al*, 2002). Calreticulin is normally found in the cytoplasma and appears on the cell surface in response to different apoptotic stimuli, such as chemotherapeutic agents (Apetoh *et al*, 2007). Apoptosis-induced complement activation differs from the classical antibody-induced activation cascade that could explain the lacking increase in C4d levels. Further *in vitro* and *in vivo* studies will be necessary to prove this assumption. Recent reports indicated that tumour cells acquire a resistance to complement-induced cell death by increasing expression of membrane-bound complement inhibitory proteins (Gorter and Meri, 1999) and/or soluble inhibitors, such as factor H (Ajona *et al*, 2007). This might further explain why complement differentially contributes to the response to chemotherapy.

Previously, it was shown that part of the cytotoxic effect of chemotherapy is mediated by the innate immune system, such as toll-like receptors (Zitvogel *et al*, 2008). Our data now suggest that the complement cascade and acute phase proteins may also be involved in this process. However, in contrast to the substantial modulation of the complement cascade and selected acute phase proteins, we found only little modulation of cytokines in the early phase of chemotherapy in our patients. Most studies in humans compared the plasma levels before therapy with the ones after its completion and hence, there are only few data available about the immediate effect of the administration of the initial dose on patients. In this study, we found minimal elevation of the interleukins IL-6, IL-10 and sICAM3 suggesting only a poor systemic cytokine response in humans. This is in contrast to several murine experiments where mice treated with etoposide or cyclophosphamide showed a rapid increase in blood levels of IL-6 that peaked at 3–6 h after administration (Elsea *et al*, 2008). Similarly, splenocytes collected from mice treated with cytarabine, cisplatin, etoposide or melphalan displayed an increase in the synthesis of several cytokines, including tumour necrosis factor-*α* within 24 h (de Vries and Singer, 2000). This provides further evidence that the chemotherapy-induced modulation of complement is not part of a classical inflammatory response, but rather an atypical stimulation.

Aside from gaining a better understanding of the immunological consequences of chemotherapy in humans, we identified early predictive markers that could enable more individualised therapy in the future. We identified two protein spots of the complement component C3 that were differentially modulated by epirubicin/docetaxel-based chemotherapy. Spot 195 is likely to represent a still non-cleaved full transcript of C3, whereas spot 529 is an isoform of its proteolytic product C3 *α*-chain. This further supports our assumption that epirubicin/docetaxel-based therapy results in activation of C3. Responders seem to show higher activation in comparison to non-responders. To date, the isoforms that we have detected can only be identified by 2D-gel electrophoresis, which renders diagnosis for a larger set of patients not possible yet. The generation of isoform-specific antibodies could change this technical obstacle. Moreover, analysis of further complement-specific or -associated markers might improve a complement-based prediction assay.

It is well established that corticosteroids can affect acute phase proteins and the complement system. With the exception of only one patient all received the glucocorticoid dexamethason-21-dihydrogenphosphate together with epirubicin and docetaxel. However, in a still ongoing study with colon carcinoma patients receiving dexamethason-21-dihydrogenphosphate, but combination with other chemotherapeutics we could not see the same changes in the plasma proteome (data not shown). Therefore, it is unlikely that this drug contributes substantially to the effects on the complement cascade described above.

It is important that our study has followed a new concept in response prediction as two different points in time were taken into consideration, allowing for the assessment of dynamic changes during chemotherapy. Previous efforts to predict chemotherapy efficiency largely relied on the assessment of predictive markers or marker sets in pre-treatment biopsies that then formed the basis to predict treatment outcome. However, assessing dynamic changes throughout the early course of neoadjuvant chemotherapy could offer the advantage that the ‘actual’ efficacy of chemotherapy is monitored *in vivo* that could provide more reliable predictive information.

In conclusion, our data suggest a significant role of the complement cascade and acute phase proteins in the host response to neoadjuvant chemotherapy with epirubicin and docetaxel in breast cancer patients. If validated in a larger series of patients, the complement system might, therefore, serve as an important future surrogate marker for therapeutic response and allow for early identification of breast cancer patients who will not profit from epirubicin/docetaxel-based neoadjuvant chemotherapy.

## Figures and Tables

**Table 1:** Patient characteristics

	**Pat. No.**	**Age**	**Type**	**T-Stage**	**N-Stage**	**ER**	**PR^a^**	**HER** **-2**	**Grading**	**Response**	**Response group**
Discovery set	1	61	Lobular	4b	+	+	+	−	2	SD	NR
	2	41	Ductal	4b	−	−	−	−	3	PR	R
	3	52	Ductal	3	−	+	−	−	3	pCR	R
	4	30	Ductal	3	+	−	−	Amplified	2	PR	R
	5	56	Lobular	3	+	+	−	−	2	PR	R
	6	74	Lobular	2	+	+	+	−	3	SD	NR
	7	51	Ductal	2	−	−	−	−	3	PR	R
	8	68	Ductal	2	−	−	−	−	3	pCR	R
	9	37	Ductal	2	−	−	−	−	3	SD	NR
	10	46	Lobular	2	+	+	−	−	3	SD	NR
	11	52	Ductal	2	+	−	−	−	3	SD	NR
	12	60	Ductal	3	+	+	−	−	1	PD	NR
Validation set	13	51	Ductal	2	−	−	−	−	3	PCR	R
	14	53	Ductal	2	+	−	−	−	3	PR	R
	15	62	Ductal	2	−	+	+	−	2	PR	R
	16	51	Ductal	2	+	−	−	−	3	SD	NR
	17	48	Ductal	3	−	+	+	Amplified	2	PCR	R
	18	40	Ductal	2	+	+	+	−	2	SD	NR
	19	30	Ductal	2	−	−	−	−	3	SD	NR
	20	59	Lobular	2	+	+	+	−	2	SD	NR
	21	66	Ductal	2	+	−	−	−	3	PR	R
	22	58	Lobular	3	+	+	−	Amplified	2	PR	R
	23	50	Lobular	2	+	+	−	−	2	SD	NR
	24	52	Ductal	2	+	+	+	−	2	PD	NR
	25	38	Ductal	2	−	+	+	−	3	SD	NR

Abbreviations: ER=oestrogen receptor; HER-2=human epidermal growth factor receptor; pCR=complete pathological remission; PD=progressive disease; NR=nonresponder; PR=partial remission; R=responder; SD=stable disease.

a Progesterone receptor.

**Table 2:** Protein spots most significantly influenced by the initial dose of epirubicin and docetaxel

**Spot ID**	**Protein**	**SwissProt ID**	**Regulation^a^**	**Function**
195	Complement component C3^b^	P01024	Up	Part of the complement system
235, 239, 242, 248, 257, 258	NI		Down	
388, 393, 397, 405, 406, 407	Inter-*α*-trypsin inhibitor heavy chain inter-*α*-trypsin inhibitor light chain	P19823 P02760	Up	Carrier of hyaluronan in serum
536	Complement component C3^c^	P01024	Up	Part of the complement system
561, 562, 576, 577	Complement component C4^c^	P0C0L4	Up	Part of the complement system
696	Complement C1r^c^	P00736	Up	Part of the complement system
929	NI		Up	
972	L-plastin	P13796	Up	Actin-binding protein (intracellular protein)
1064, 1075^d^	*α*-1-antichymotrypsin^c^	P01011	Up	Physiological function is unclear, can inhibit formation of active angiotensin-2
1088^d^, 1100^d^, 1120, 1132^d^, 1133^d^, 1136^d^, 1160^d^, 1162^d^	*α*-2-HS-glycoprotein^c^	P02765	Down	Promotes endocytosis and possesses opsonic properties
1335	NI		Up	
1340	Complement component C4	P0C0L4	Up	Part of the complement system

Abbreviation: NI=not identified.

Note: The paired Student's *t*-test was corrected for multiple testing using the false discovery rate algorithm according to Benjamini and Hochberg (1995).

a All proteins fulfill the conditions of *P*<0.05 and minimal mean change in expression level of 30%.

b Identification only by 2D-western blotting.

c Identification was confirmed by 2D-western blotting using specific antibodies.

d The position on the gel allowed unambigous identification.

**Table 3:** Influence of the chemotherapeutic initial dose on cytokines and complement components C3, C4, as well as the activation fragment C4d

	**Before initial dose**			**After initial dose**			**Paired Student's *t*-test**
	**Mean**	**s.d.**	**Units**	**Mean**	**s.d.**	**units**	***P*-value**
*Cytokine panel*							
*Discovery set (n*=*12)*							
TNF*α*	ND		pg ml^−1^	ND		pg ml^−1^	—
TNF*β*	ND		pg ml^−1^	ND		pg ml^−1^	—
IFN*γ*	ND		pg ml^−1^	ND		pg ml^−1^	—
IL-1*β*	ND		pg ml^−1^	ND		pg ml^−1^	—
IL-2	27.7	29.4	pg ml^−1^	24.6	36.4	pg ml^−1^	0.491
IL-4	ND		pg ml^−1^	ND		pg ml^−1^	—
IL-5	ND		pg ml^−1^	ND		pg ml^−1^	—
IL-6	1.0	0.01	pg ml^−1^	4.5	1.6	pg ml^−1^	0.003
IL-8	2.6	17.6	pg ml^−1^	23.1	30.7	pg ml^−1^	0.268
IL-10	5.1	1.0	pg ml^−1^	8.5	3.5	pg ml^−1^	0.001
IL-12p70	ND		pg ml^−1^	ND		pg ml^−1^	—
sE-selectin	86.7	52.5	ng ml^−1^	88.8	55.5	ng ml^−1^	0.179
sICAM-1	453.5	177.4	ng ml^−1^	413.1	146.8	ng ml^−1^	0.181
sICAM-3	87.6	23.8	ng ml^−1^	64.1	20.7	ng ml^−1^	0.011
sPECAM-1	200.4	93.5	ng ml^−1^	139.4	61.7	ng ml^−1^	0.069
sP-selectin	428.5	182.5	ng ml^−1^	405	130.9	ng ml^−1^	0.178
sVCAM-1	420.8	622.4	ng ml^−1^	468.4	259.7	ng ml^−1^	0.288
*Complement factors*							
*Discovery set (n*=*12)*							
C3	144.2	15.40	mg per 100 ml	127.9	17.3	mg per 100 ml	0.002
C4	30.4	6.00	mg per 100 ml	24.5	6.8	mg per 100 ml	<0.001
C4d	3.6	1.40	μg ml^−1^	2.6	1.0	μg ml^−1^	0.007
							
*Validation set (n*=*13)*							
C3	124.6	16.50	mg per 100 ml	118.1	19.3	mg per 100 ml	0.011
C4	27.0	5.90	mg per 100 ml	23.5	5.3	mg per 100 ml	0.019
C4d	2.8	1.00	μg ml^−1^	2.2	0.8	μg ml^−1^	0.004

Abbreviation: ND=not detectable.

**Table 4:** Isoforms of complement components C3 and C4 correlating with response to chemotherapy

		**Discovery set**				**Validation set**				**Overall**	
		**Mean change in expression after initial dose**				**Mean change in expression after initial dose**				**Δ^b^**	***Student's t*-test**
**Complement factor^a^**	**spot-ID**	**non-resp (*n*=6)**	**Resp (*n*=6)**	**Δ^a^**	***t*-test**	**non-resp (*n*=7)**	**resp (*n*=6)**	**Δ^a^**	***t*-test**	**(*n*=25)**	
*C3*											
Group 1	195	0.415	0.138	−0.277	0.045	0.209	−0.167	−0.376	0.034	−0.309	0.013
*α*1	465	−0.110	0.075	0.185	0.049	0.010	0.140	0.130	0.128	0.150	0.026
*α*2	529	−0.067	0.177	0.244	0.001	−0.061	0.304	0.365	0.054	0.304	0.005
*α*2	534	0.018	0.427	0.409	0.040	−0.114	−0.025	0.089	0.289	0.209	0.070
											
*C4*											
*α*1	559	−0.056	0.247	0.302	0.028	0.108	0.189	0.081	0.298	0.188	0.037
*α*1	561	0.118	0.514	0.397	0.027	0.085	0.218	0.133	0.307	0.266	0.053
*α*2	676	−0.048	0.072	0.120	0.026	0.051	0.210	0.159	0.170	0.135	0.066

a According to Figure 1: C3 *α*1=group 2; C3 *α*2=group 3; C4 *α*1=group 1; C4 *α*2=group 2.

b Difference between non-responder and responder.
