# Supplementary material for: Modulation of plasma complement by the initial dose of epirubicin/docetaxel therapy in breast cancer and its predictive value
Source: Br J Cancer. 2010 Sep 28;103(8):1201–8. doi: 10.1038/sj.bjc.6605909 (PMC2967072; doi:10.1038/sj.bjc.6605909)
Supplement: Supplementary Data S1–S2 [file 6605909x1.pdf]

Influence of chemotherapy

Discovery set (n=12)

**Spot-ID**	**before initial dose**		**after initial dose**		**paired t-test** [(1)]
	mean [(2)]	SD	mean [(2)]	SD	p-value
195	0,891	0,183	1,155	0,230	0,033
235	1,075	0,201	0,807	0,170	< 0.01
239	1,112	0,270	0,797	0,226	< 0.01
242	1,113	0,301	0,764	0,247	< 0.001
248	1,142	0,305	0,806	0,248	< 0.01
257	1,159	0,309	0,815	0,237	< 0.01
258	1,125	0,311	0,823	0,253	< 0.01
388	0,844	0,172	1,134	0,299	< 0.01
393	0,813	0,172	1,184	0,362	0,014
397	0,818	0,206	1,220	0,359	< 0.01
405	0,834	0,257	1,201	0,376	< 0.01
406	0,852	0,270	1,181	0,370	< 0.01
407	0,893	0,305	1,146	0,383	< 0.01
536	0,927	0,230	1,291	0,542	0,028
561	0,992	0,379	1,307	0,503	0,018
562	0,907	0,273	1,244	0,424	< 0.01
576	0,887	0,242	1,192	0,431	0,018
577	0,833	0,220	1,216	0,518	< 0.01
696	0,896	0,146	1,203	0,232	< 0.01
929	0,848	0,253	1,070	0,275	0,012
972	0,847	0,166	1,371	0,524	< 0.01
1064	1,037	0,306	1,376	0,456	< 0.01
1075	1,031	0,301	1,413	0,527	< 0.01
1088	1,183	0,511	0,858	0,401	< 0.01
1100	1,262	0,625	0,892	0,509	< 0.01
1120	1,276	0,607	0,841	0,527	< 0.01
1132	1,249	0,613	0,775	0,521	< 0.01
1133	1,209	0,660	0,682	0,536	< 0.01
1136	1,300	0,680	0,927	0,576	< 0.01
1160	1,156	0,994	0,583	0,428	< 0.01
1162	1,209	0,956	0,571	0,338	< 0.01
1335	0,814	0,311	1,051	0,341	0,020
1340	0,807	0,288	1,047	0,370	0,014

[(1)] The paired t-test was corrected for multiple testing using the false discovery rate algorithm according to Benjamini and Hochberg (1995)

[(2)] Standardized abundance

Michlmayr et al.; Supplementary Data, S2

Spot ID	Protein name	Acc. no.	PMF	MS/MS	
			Seq.cov. %	Peptides	sequence
393-407	Inter-α-trypsin inhibitor heavy chain	P19823	9 (MASCOT, ProFound)<br>13 (Aldente)	1468.74<br>1337.65	AHVSFKPTVAQQR<br>FYNQVSTPLLR
	Inter-α-trypsin inhibitor light chain	P02760	13 (MASCOT)<br>31 (ProFound)<br>22 (Aldente)	1682,79	TVAAC(CAM)NLPIV-RGPC(CAM)R
536	Complement component C3b	P01024	39 (MASCOT)<br>21 (ProFound)<br>20 (Aldente)	n.i.	
561	Complement component C4	P0C0L4	n.i.	983,79	QGSFQGGFR
562	Complement component C4	P0C0L4	19 (MASCOT)<br>27 (ProFound)<br>18 (Aldente)	n.i.	
576	Complement component C4	P0C0L4	12 (Aldente)	n.i.	
577	Complement component C4	P0C0L4	9 (ProFound)<br>12 (Aldente)	n.i.	
696	Complement C1r precursor	P00736	13 (MASCOT)<br>16 (ProFound)<br>14 (Aldente)	n.i.	
972	L-plastin	P13796	28 (AMSCOT)<br>26 (ProFound)<br>18 (Aldente)	1676.05<br>1576.93<br>1584.93	FSLVGIGGQDLNEGNR<br>WANYHLENAGCNK<br>VYALPEDLVEVNPK
1064	α-1-antichymotrypsin	P01011	10 (Mascot)		
1120	α-2-HS-glycoprotein	P02765	8 (MASCOT)<br>9 (ProFound)		
1340	Complement component C4	P0C0L4	6 (MASCOT)<br>6 (ProFound)<br>55 (Aldente)	972.55<br>1191.67	DKGQAGLQR<br>LGQYASPTAKR
